# Patient-reported outcome measures for monitoring primary care patients with depression (PROMDEP): study protocol for a randomised controlled trial

**DOI:** 10.1186/s13063-020-04344-9

**Published:** 2020-05-29

**Authors:** Tony Kendrick, Michael Moore, Geraldine Leydon, Beth Stuart, Adam W. A. Geraghty, Guiqing Yao, Glyn Lewis, Gareth Griffiths, Carl May, Rachel Dewar-Haggart, Samantha Williams, Shihua Zhu, Christopher Dowrick

**Affiliations:** 1 Primary Care, Population Sciences, and Medical Education, University of Southampton, Aldermoor Health Centre, Southampton, SO16 5ST UK; 2 Department of Health Sciences, University of Leicester, George Davies Centre, University Road Leicester, Leicester, LE1 7RH UK; 3 Division of Psychiatry, Faculty of Brain Sciences, University College London, 6th Floor, Maple House, 149 Tottenham Court Rd, London, W1T 7NF UK; 4 Southampton Clinical Trials Unit, University of Southampton, Southampton General Hospital, Tremona Road, Southampton, SO16 6YD UK; 5 Department of Health Services Research and Policy, Faculty of Public Health and Policy, London School of Hygiene and Tropical Medicine, 15-17 Tavistock Place, London, WC1H 9SH UK; 6 Institute of Psychology Health and Society, University of Liverpool, Liverpool, L69 3GL UK

**Keywords:** Depression, Primary care, Monitoring, Patient-reported outcome measures, PHQ-9

## Abstract

**Background:**

Benefits to patients from reduced depression have been shown from monitoring progress with patient-reported outcome measures (PROMs) in psychological therapy and mental health settings. This approach has not yet been researched in the United Kingdom for primary care, which is where most people with depression are treated in the United Kingdom.

**Methods:**

This is a parallel-group cluster randomised trial with 1:1 allocation to intervention and control. Patients who are age 18+ years, with a new episode of depressive disorder/symptoms, meet the inclusion criteria. Patients with current depression treatment, comorbid dementia/psychosis/substance misuse/suicidal ideas are excluded. The intervention includes the Administration of Patient Health Questionnaire (PHQ-9) as a PROM within 2 weeks of diagnosis and at follow-up 4 weeks later. General practitioners are trained in interpreting scores and asked to take them into account in their treatment decisions. Patients are given written feedback on scores and suggested treatments. The primary outcome measure is Depression on the Beck Depression Inventory BDI-II at 12 weeks. Secondary outcomes include BDI-II at 26 weeks, changes in drug treatments and referrals, social functioning (Work & Social Adjustment Scale) and quality of life (EQ-5D) at 12 and 26 weeks, service use over 26 weeks (modified Client Services Receipt Inventory) to calculate NHS costs, and patient satisfaction at 26 weeks (Medical Informant Satisfaction Scale). The sample includes 676 total participants from 113 practices across three centres. Randomisation is achieved by computerised sequence generation. Blinding is impossible given the nature of the intervention (self-report outcome measures prevent rating bias). Differences at 12 and 26 weeks between intervention and controls in depression, social functioning and quality of life are analysed using linear mixed models, adjusted for socio-demographics, baseline depression, anxiety, and clustering, while including practice as a random effect. Patient satisfaction, quality of life (QALYs) and costs over 26 weeks will be compared between arms. Qualitative process analysis includes interviews with 15–20 GP/NPs and 15–20 patients per arm to reflect trial results and implementation issues, using Normalization Process Theory as a theoretical framework.

**Discussion:**

If PROMs are helpful in improving patient outcomes for depression even to a small extent, then they are likely to be good value for money, given their low cost. The benefits could be considerable, given that depression is common, disabling, and costly.

**Trial registration:**

ISRCTN no: 17299295. Registered 1st October 2018.

**Administrative information**

**Table:** 

Title {1}	Patient-reported outcome measures for monitoring primary care patients with depression: PROMDEP randomised controlled trial
Trial registration {2a and 2b}.	Trial registration: ISRCTN no: 17299295, registered 1st October 2018
Protocol version {3}	Version 1.4 Dated 4th January 2019
Funding {4}	National Institute for Health Research (NIHR) Health Technology Assessment (HTA) programme. Ref: HTA 17/42/02. Funding acknowledgement: The views expressed are those of the author(s) and not necessarily those of the NIHR or the Department of Health and Social Care.
Author details {5a}	Tony Kendrick, Professor of Primary Care, email: A.R.Kendrick@soton.ac.uk Beth Stuart, Associate Professor, bls1@soton.ac.uk Adam WA Geraghty, Senior Research Fellow, A.W.Geraghty@soton.ac.uk Geraldine Leydon, Professor of Medical Sociology, G.M.Leydon@soton.ac.uk Michael Moore, Professor of Primary Health Care Research, M.V.Moore@soton.ac.uk Rachel Dewar-Haggart, Senior Research Assistant, R.V.Dewar-Haggart@soton.ac.uk Samantha Williams, Senior Research Assistant, S.J.Williams@soton.ac.uk Shihua Zhu, Health Economist, s.zhu@soton.ac.uk **Primary Care, Population Sciences, and Medical Education, University of Southampton, Aldermoor Health Centre, Southampton SO16 5ST.** Carl May, Professor of Medical Sociology, carl.may@lshtm.ac.uk **Department of Health Services Research and Policy, Faculty of Public Health and Policy, London School of Hygiene and Tropical Medicine, 15–17 Tavistock Place, London WC1H 9SH.** Glyn Lewis, Professor of Epidemiological Psychiatry, glyn.lewis@ucl.ac.uk **Division of Psychiatry, Faculty of Brain Sciences, University College London, 6th Floor, Maple House, 149 Tottenham Court Rd, London, W1T 7NF.** Guiqing Lily Yao, Professor of Health Economics, gy38@leicester.ac.uk **Department of Health Sciences, University of Leicester, George Davies Centre, University Road Leicester, LE1 7RH.** Gareth Griffiths, Professor of Clinical Trials, G.O.Griffiths@soton.ac.uk **Southampton Clinical Trials Unit, University of Southampton and University Hospitals Southampton NHS Foundation Trust, Southampton General Hospital, Tremona Road, Southampton, SO16 6YD.** Christopher Dowrick, Professor of Primary Medical Care, cfd@liverpool.ac.uk **Institute of Psychology Health and Society, University of Liverpool, Liverpool L69 3GL.**
Name and contact information for the trial sponsor {5b}	Dr Alison Knight, Head of Research Governance, Research Governance Office, University of Southampton, Room 4079, Building 37, Highfield Campus, Southampton SO17 1BJ. Tel: 0238059 5058. Fax: 0238059 5781. Email: rgoinfo@soton.ac.uk
Role of sponsor {5c}	The study sponsor and funder (National Institute for Health Research) were not involved in the study design; writing of the protocol paper; or the decision to submit the paper for publication.

## Background

### Background and rationale {6a}

England, like other countries, has seen big increases in antidepressants and psychotherapy for depression since the early 1990s, yet the prevalence of depression has not declined but actually has increased slightly. One of the main reasons is a lack of application of evidence-based treatments to those who would benefit– referred to as the ‘quality gap’ [1].

NICE guidelines recommend different treatments for more severe depression than for less severe depression [2]. However, general practitioners (GPs), who treat more than 80% of cases in primary care, are often inaccurate in their global clinical assessments of depression severity, and so treatment is not targeted to patients most likely to benefit [3]. Some patients receive treatment they do not need (medicalising self-limiting illness and exposing them to side effects) and others do not get the treatment they do need, significantly contributing to the ‘quality gap’. A systematic review concluded that many false diagnoses occur, as well as missed cases, which could be improved by reassessment of individuals who might have depression [4]. As a result, the National Institute for Health and Care Excellence (NICE) recommends practitioners consider using depression symptom questionnaires as validated measures of severity at diagnosis and follow-up, to inform and evaluate treatment [2].

Questionnaire use was incentivised in the GP contract from 2006 to 2013. However, since those payments stopped, most GPs prefer not to use them, saying they intrude in consultations and undermine their autonomy. Some doubt their validity, preferring to use their own judgement to assess severity and response to treatment [5].

A Cochrane review of using PROMs in treating common mental health disorders (CMHDs) including depression found some evidence of benefit in psychotherapy and specialist mental health settings, but the research was generally of low quality, and hardly any research had been done in primary care [6]. More research is therefore required, particularly in UK primary care, where most patients are treated if they are treated at all.

Depression is common and costly. The 1-week prevalence among adults in the United Kingdom is 11.1%, including 3.3% major depression and 7.8% mixed depression and anxiety [7]. Depression can lead to chronic disability, poor quality of life, suicide, and high service use and costs. The King’s Fund estimated that 1.45 million people will have depression in England by 2026, and annual costs for care, social services and lost employment will be £12.2 billion [8]. If using severity measures improves the targeting of treatment and outcomes for depression even to a modest extent, they are likely to be cost-effective given their low cost, and the benefits at a population level would be considerable in public health terms, given the high costs of depression.

Depression symptom questionnaires are an example of patient-reported outcome measures (PROMs), which have been promoted to increase patient involvement in their own care [9], and research shows that patients value the use of questionnaire severity measures to confirm their diagnosis and monitor their progress [5].

Observational research suggests depression questionnaires can also improve the process of care for patients. Following NICE guidance, from 2006 to 2013, the GP contract Quality and Outcomes Framework (QOF) paid GPs to use symptom questionnaires to assess depression severity at diagnosis of a new episode. Questionnaire assessments at follow-up were also rewarded in the QOF from 2009 to 2013. Our previous observational research conducted in the year following the introduction of the QOF incentivisation of questionnaire use found that patients valued the use of them to confirm their diagnosis and monitor their progress, and some GPs also valued them for monitoring patients [5]. Importantly, treatment was better targeted. The likelihood of antidepressant treatment or referral to psychology was found to be significantly associated with higher questionnaire scores at diagnosis [10], and at follow-up, decisions to change treatment were significantly associated with the lack of improvement in scores [11].

The use of questionnaires was disliked by some GPs however, who said the questionnaires intruded in consultations and undermined their autonomy. Some doubted their validity, preferring to use their own judgement to assess severity and response to treatment [5]. In response to these criticisms, NICE commissioned a review [12] which concluded the evidence was not strong enough to require their use in QOF depression indicators. Currently, the QOF rewards reviews 10–35 days after diagnosis, but questionnaires are optional and not required to receive payments.

However, a recent time series analysis of GP prescribing data shows the QOF depression indicators were associated with a subsequent reduction in antidepressant prescribing for first-ever episodes of depression, which is in line with NICE guidance not to give antidepressants for mild depression [13]; therefore, rewarding questionnaire use should be seriously reconsidered.

Routine outcome monitoring with questionnaire measures of depression severity takes place in the NHS Improving Access to Psychological Therapy (IAPT) psychological treatment services and has been shown to improve the efficiency of care in that setting [14]. However, only 15% of CMHD patients are treated by the IAPT programme [15], so more research into the potential benefits of routine outcome monitoring with depression symptom severity measures is required in UK primary care, where most patients are treated, if they are treated at all.

Systematic reviews of PROMs for depression have found some evidence of benefit for patients treated in mental health [16] and psychological therapy settings [17], but a recent Cochrane review found little research had been done in primary care [6]. We carried out a feasibility randomised controlled trial (RCT) of PROMs for depression in UK primary care [18]. We tested individual patient and cluster randomisation of 47 adults with new episodes—22 for the intervention and 25 for the control—in nine practices. Three PROMs were administered following diagnosis and again 10–35 days later: the Patient Health Questionnaire PHQ-9 [19], the Distress Thermometer analogue scale [20] and the PSYCHLOPS problem profile [21]. Feedback of scores to patients was left to the practitioners. Mean BDI-II score at 12 weeks was lower among intervention group patients than controls by 5.8 points (95% CI-11.1, − 0.5), adjusted for baseline differences and practice [18]. Social functioning scores were not significantly different. At 26 weeks, no significant differences existed in symptoms, social functioning, quality of life or costs, but the mean satisfaction score was lower among intervention patients by 22.0 points (− 40.7,-3.29). Qualitative interviews suggested this was because patients were disappointed when their GPs did not use PROM scores to inform treatment. Some GPs were not convinced the PROMs were useful and wanted more guidance on treatment actions in response to the scores [18].

We concluded PROMs might improve outcomes, even if they do not always inform management, in line with the findings of a similar trial using the PHQ-9 in the USA [22]. Patients can feel more involved in their care and more motivated to adhere to treatment and follow-up [23, 24]. Primary care patients in Sweden monitored with the Montgomery-Asberg rating scale were more likely to adhere to antidepressants, but no improvements in outcome were observed in that study [25], so findings are variable and more research needed.

## Objectives {7}

The objectives of the study are as follows:

1. To carry out a cluster-randomised, controlled, parallel group trial that will compare (i) getting patients to complete the PHQ-9, which is used as a patient-reported outcome measure (PROM) in their consultations with General Medical Practitioners (GPs) or Nurse Practitioners (NPs) treating them for depression, with (ii) usual Practitioner care, uninformed by PHQ-9 scores
2. To motivate and train participating practitioners to reflect on the best use of the PHQ-9, thereby improving the practitioner’s capability to interpret symptom scores, taking into account patients’ responses to open-ended global enquiries, their level of functioning, past history, and social context, including life events and difficulties
3. To provide patients in the intervention arm with written feedback on their PHQ-9 scores, including a ‘traffic light’ indication of the level of severity of their depression, a 100-manikin representation of the proportion of people in the population with that level of depression, and a brief list of evidence-based treatments relevant to the level of severity, which they will be asked to discuss with their GP/NP
4. To follow up participants for 26 weeks, with research assessments at 12 and 26 weeks
5. To determine the primary outcome of depressive symptoms on the Beck Depression Inventory, 2nd edition (BDI-II), at 12 weeks follow-up
6. To examine secondary outcomes including depressive symptoms on the BDI-II at 26 weeks, and social functioning, quality of life, and changes in drug treatment and referrals, at both 12 and 26 weeks
7. To measure service use and costs over the 26-week follow-up period and perform cost-effectiveness and cost-utility analyses based upon the results of the trial
8. To carry out a qualitative process analysis to explore participants’ reflections on the conduct of the trial, and the potential for implementing the use of PROMs in practice, using. interviews with 15–20 practitioners and 15–20 patients, which will be carried out using Normalization Process Theory [26] as a theoretical framework

## Trial design {8}

The study is a parallel group, cluster-randomised, controlled, superiority trial, with patients clustered by participating practices, and 1:1 allocation of practices to intervention and control groups. We chose a cluster-randomised design after a feasibility study that showed that randomising patients individually within practices risks contamination between study arms.

## Methods/Design

### Study setting {9}

The setting is UK primary care, recruiting general practices around three sites: the University of Southampton, University of Liverpool, and University College London (UCL). The full list of sites can be obtained by email from study administrative assistant Sophie Johnson at promdep@soton.ac.uk.

### Eligibility criteria {10}

The target population is patients aged 18 or more years, diagnosed by a GP or NP with a new episode of depression disorder or depressive symptoms. A new episode means no diagnosis or treatment within the previous 3 months. The inclusion criteria are adult patients seen in the practice within the last 2 weeks and assigned medical record Read codes by GPs or NPs for new presentations with diagnoses or symptoms of depression. No upper age limit will be implemented, and patients will not be excluded for coexisting physical health problems. Patients will be excluded if they are already being treated for depression, or if they have comorbid dementia, psychosis, or substance misuse (as a main problem). Patients will also be excluded if they have significant suicidal thoughts requiring possible urgent referral to specialist mental health care (see below).

### Who will take informed consent? {26a}

Patients identified as potentially eligible in clinic consultations will be given an information sheet by hand, together with a reply slip and a Freepost envelope, and they will be asked to contact the study team if they wish to take part after they have had time to consider. To avoid selection bias by the GP/NP, patients presenting with a new episode of depressive symptoms or disorder will also be identified through weekly searches of practice medical records databases for the identification of patients who were not selected by the GP/NP. Patients identified through this method will be mailed an information sheet about the study by the practice and asked to contact the study team if they wish to take part, or to decline, again using a reply slip and a Freepost envelope, after they have had time to consider. If they do not respond, the research team will have no knowledge of them, maintaining patient confidentiality, and this will not prejudice their future treatment. If patients do respond positively to either approach, a member of the research team will then contact them, screen them by telephone for any exclusion criteria, and arrange to see them face to face for a baseline visit if they are eligible. At the baseline visit, the researcher will go over the patient information sheet again and seek formal written consent.

### Additional consent provisions for collection and use of participant data and biological specimens {26b}

This trial does not involve collecting biological specimens for storage.

### Interventions

#### Intervention description {11a}

The intervention consists of getting patients to complete the Patient Health Questionnaire (PHQ-9), which measures depression symptoms, for use as a patient-reported outcome measure (PROM) in their consultations with GPs (or Nurse Practitioners, NPs) treating them for depression. The PHQ-9 will be completed by participating patients as soon as possible after diagnosis and then again at a follow-up consultation 10–35 days after that (this follow-up time period has been chosen, as it is the interval laid down for financially incentivised follow-up assessments in the GP contract quality outcomes framework QOF). Patients will be given feedback on the meaning of their symptom score and possible treatment options to discuss with the practitioner. Practitioners will be trained in the interpretation of symptom scores in the context of the patient’s life situation, and they will be trained in further assessment to inform their treatment decisions.

The PHQ-9 is a nine-question self-report measure of depression symptoms that takes approximately three minutes to complete [19]. It asks about the American Psychiatric Association’s Diagnostic and Statistical Manual (DSM) nine diagnostic symptoms of major depressive disorder and scores on each symptom range from 0 (not at all) to 3 (nearly every day). Total scores are categorised into minimal or no (0–4), mild (5–9), moderate (10–14) and severe (15–27) depression. It was developed and originally validated against diagnostic interviews in the USA and can be downloaded free of charge from www.depressionprimarycare.org/clinicians/toolkits/materials/forms/phq9/questionnaire/. Pfizer owns the copyright but does not charge for its use in clinical practice or research [27].

We will provide patients in the intervention arm with written feedback on their PHQ-9 scores, including a ‘traffic light’ indication of the level of severity of their depression, a 100 manikin infographic of the proportion of people in the population with their level of depression and a brief indication of possible evidence-based treatments relevant to the level of severity, which they will be asked to discuss with their GP/NP. PHQ-9 sum scores range from 0 to 27, with scores of 0–4, 5–9, 10–14 and 15+ representing ‘probable minimal or no depression’, ‘mild’, ‘moderate’ and ‘moderately severe to severe’ depression symptom levels, respectively. These will be fed back to patients as one of four severity categories: green (70% of population on the 100 manikin infographic), yellow (20%), orange (8%) and red (2%), respectively. The patient feedback proformas were derived from similar ones used in the DEPSCREEN-INFO study of depression screening with patient-targeted feedback in cardiology [28].

#### Explanation for the choice of comparators {6b}

Control practice patients will not complete the PHQ-9. They will receive usual practitioner care for new episodes of depression and complete research outcome measures as part of the trial but will not be given feedback on the results.

#### Criteria for discontinuing or modifying allocated interventions {11b}

No special criteria have been developed for discontinuing or modifying the allocated interventions.

#### Strategies to improve adherence to interventions {11c}

Many GPs in UK practice are familiar with the PHQ-9, as it was the most frequently used PROM in practices in the period (2006–13) during which the use of depression symptom questionnaires was incentivised by the QOF [10]. However, some GPs doubt the validity of the PHQ-9, preferring to use their own judgement to assess severity and response to treatment [5]. The PHQ-9 meets minimum standards for a PROM [29] in terms of established validity in UK primary care [30] and sensitivity to change in response to treatment, at least at the group level [31]. It was recommended for use by the PROM Research Group at the Oxford Department of Public Health as having evidence to support its use and being broadly acceptable [32].

The severity categories of the PHQ-9 can be criticised, as a score of 5–9 (‘mild depression’) is found in approximately 20% of the population, but is not usually associated with significantly impaired functioning, so labelling people scoring 5–9 as having depression may be counterproductive, in that treatment is not usually indicated, and the label itself may make patients feel worse about themselves. The cut-off of a score of 10 for moderate depression, as the threshold for offering treatment, has also been questioned, and studies have suggested a score of 12 may be a more valid threshold [10]. The PHQ-9 tends to put more people in the ‘moderate depression’ category than other PROMs such as the Hospital Anxiety and Depression Scale for example [10, 33].

In any case, an initial high PHQ-9 score by itself, whether above 10 or above 12, does not suffice to indicate the need for antidepressant drug treatment or referral for psychological therapy because patients vary greatly in their propensity to acknowledge symptoms when asked. Recent qualitative research led by co-applicants Dowrick and Lewis suggests that the PHQ-9 is not exhaustive in its list of symptoms and not all patients find it straightforward to complete, so it may miss symptoms that are meaningful to patients (e.g., changes in libido, social withdrawal, interpersonal difficulties), and underestimate their intensity [34]. Some patients consciously underreport their symptoms to try to reduce them through positive affirmations, while others overreport them to emphasise they want help. Consequently, as many as half of patients rated with the PHQ-9 may have a mismatch with how they describe their overall condition at baseline, as well as their progress over time, when asked global, open-ended questions [34].

Symptom scores are therefore quite individual, and the baseline level has to be interpreted in light of the impact of the symptoms on the person’s functioning at home, at work and in relationships. The recent qualitative research suggests that the PHQ-9 should not be used as a standalone tool but should preferably be used in conjunction with an open-ended enquiry such as ‘how are you feeling in yourself?’ [34], as a better measure of the person’s unique ongoing experience of their depression. Additionally, within-person changes in individuals’ PHQ-9 scores between the first and second consultation are only limited indicators of whether patients are improving, not improving or getting worse and therefore need to be supplemented with a global enquiry such as ‘how are you feeling in comparison to when I last saw you?’ along with an update on their life circumstances [34]. Where mismatches exist between changes in patient scores and global ratings of change, practitioners need to take particular care when interpreting the results of the PHQ-9.

Our feasibility study suggested that GPs’ discussion of the PHQ-9 scores with patients and their use of them to inform treatment were suboptimal, affecting both their own perception of the measure, and patients’ satisfaction with the care they received [18]. To change practitioner behaviour in the proposed trial, we will implement 2 hours of structured training. By triangulating our qualitative feasibility findings with behavioural theory [35], we determined the need for the training to focus primarily on GP’s reflective motivation (e.g., beliefs about the usefulness of PROMS) and psychological capability (e.g., knowledge and understanding to apply PROMS effectively). These constructs are drawn from the ‘COM-B’ system of behaviour (referring to Capability, Opportunity, Motivation and Behaviour) [35]. The COM-B system is used widely in behaviour change research and focuses on necessary antecedents for voluntary behaviour to occur. Participating GPs will therefore be given up to 2 hours of training either face to face on their practice premises or on-line, including written material beforehand (informed by the NICE guidelines), a PowerPoint presentation, case vignettes, and questions for them to answer to show they have understood the training.

To get GPs/NPs to reflect on the value of the use of the measure, the training will focus on evidence that patients do value using PROMs and can benefit from being more involved in their own care even if the scores do not alter treatment decisions. We will address GP/NP concerns around the validity of the PHQ-9 by acknowledging individual differences in patient response set, and advising them to combine more global open-ended questions with the questionnaire measure. GPs will thus be trained in interpreting individual PHQ-9 scores along with how to ask open-ended questions and explore the patient’s life context; these GPs will be asked to take this information into account in their treatment decisions.

#### Relevant concomitant care permitted or prohibited during the trial {11d}

Practitioners in both the intervention and control groups will be advised that best practice in treating depression is not to start treatment at the consultation at which symptoms of a new episode are presented by the patient, unless they think it is absolutely indicated in their clinical judgment. This is because a significant proportion of patients will improve without treatment within 2–3 weeks, having had their problems acknowledged and having received general advice about the nature and course of depression. We are interested in this study with the use of the PHQ-9 when deciding on initial treatment, as well as follow-up monitoring, so we prefer treatment not be started before the baseline assessment in both groups and before the first PHQ-9 questionnaire has been administered by the researcher in the intervention group. In the feasibility study, this baseline assessment was carried out on average 10 days (range 1–38 days) from receiving the patient’s reply slip, so completion of this baseline assessment within 2 weeks of the patient’s first presentation should be possible in most cases.

However, patients recruited either opportunistically or via the weekly searches possibly will have been started on treatment at the consultation, when they first presented with a new episode, if treatment cannot be postponed in the judgement of the treating practitioner. We will record whether treatment has already started at the baseline assessment.

#### Provisions for post-trial care {30}

No harm or compensation is anticipated for trial participation.

### Outcomes {12}

The primary outcome is the symptom score on the Beck Depression Inventory second edition BDI-II [36] for the current level of depression at the 12-week follow-up. Secondary outcomes are the BDI-II score at 26 weeks, anxiety on the GAD-7 measure of generalised anxiety disorder [37], and scores at both 12 and 26 weeks on the Work & Social Adjustment Scale [38] for social functioning, the EuroQol 5-item 5-level (EQ-5D) questionnaire for quality of life [39] and the number and amounts of drug treatments and referrals for depression over the 26 weeks of total follow-up, as determined using a modified version of the Client Service Receipt Inventory [40] to calculate NHS costs. In addition, a modified version of the Medical Informant Satisfaction Scale MISS [41] will be administered at 26 weeks to measure patient satisfaction over the follow-up period.

The Beck Depression Inventory, second edition BDI-II is a 21-item self-report instrument that uses DSM-IV criteria [36]. It has been established as a valid and reliable instrument for depression screening in the general population [36, 42] and is widely used in depression trials. It takes approximately 5 minutes to complete. Each item is scored from 0 to 3, and a total score of 0–13 is considered minimal range, 14–19 is mild, 20–28 is moderate, and 29–63 is severe.

The Work and Social Adjustment Scale (WASA) assesses problems in functioning with work, home management, social leisure activities, private leisure activities and family and relationships, all on 0 to 8 scales [38]. It has been shown to be a sensitive, reliable and valid measure of impaired functioning and is used routinely in IAPT psychological therapy settings as well as in research studies in a variety of settings.

The EuroQol-5D (EQ-5D)-5 L measure of health-related quality of life [39] is the measure favoured by NICE in determining cost-effectiveness when developing its clinical guidelines. The EQ-5D includes five dimensions: mobility, self-care, usual activities, pain/discomfort, and anxiety/depression, each scored on five levels. Health states are converted into a single summary index by applying weights to each level in each dimension derived from the valuation of EQ-5D health states in adult general population samples [43]. The EQ-5D measure of patient utility will be used to determine changes in quality adjusted life years (QALYs) for the health economics evaluation.

Costs will be calculated from responses to the Client Service Receipt Inventory CSRI [40], modified specifically for the study. A review of participating patients’ digital medical records will also be carried out by practice staff after the 26-week follow-up, to augment questionnaire measurement of health and social service resource use using the modified CSRI.

The 29-item ‘Medical Interview Satisfaction Scale’ (MISS-29) was developed in the USA to assess patient satisfaction with individual doctor-patient consultations and has been shown to be valid and reliable in UK primary care [41]. We will adapt it to rate patient satisfaction at the 26-week follow-up, asking patients to look back over their consultations with GPs/NPs over the entire 26-week period.

#### Participant timeline {13}



### Sample size {14}

We need a sample large enough to detect a difference between arms at follow-up of the minimal clinically important score (MCID) on the primary outcome: the Beck Depression Inventory 2nd edition (BDI-II).

Button et al. [44] used data collected from three randomised controlled trials (*n* = 1039) for the management of depression and compared improvement on a ‘global rating of change’ question with changes in BDI-II scores. They used general linear modelling to explore baseline dependency, assessing whether MCID is best measured in absolute terms (i.e., difference) or as percentage reduction in scores from baseline (i.e., ratio). The modelling indicated that MCID is best measured on a ratio scale as a percentage reduction of score, and an MCID of a 17.5% reduction from baseline was identified from receiver operator characteristics analyses as the optimal threshold above which individuals reported feeling ‘better’ [44].

In the PROMDEP feasibility trial, we found the mean BDI-II score at baseline was 24.0, and the standard deviation (SD) was 10.0 [18]. At the 12-week follow-up, based on the results of the feasibility study, we anticipate a mean of 14.0 in the intervention group and 17.0 in the control group. This gives a mean difference of 3.0 on the BDI-II, which is an effect size of 0.3 SDs and agrees with the findings of Knaup et al’s systematic review for the expected effects of combined practitioner and patient feedback of PROMs [16]. The difference of 3.0 points is 17.6% of the control group’s score of 17.0 at 12 weeks, and therefore, this score is just above the MCID for the BDI-II [44]. The anticipated potential benefit would therefore be small but clinically significant.

We aim to recruit a mean of six patients per practice. We assume an intra-cluster correlation coefficient (ICC) of 0.03 (from the feasibility study). At the level of 5% significance, to have 90% power to detect a difference between 14.0 and 17.0 on the BDI-II we need 235 patients analysed per group. Given a cluster size of six, the cluster design effect will be 1.15, meaning we need 270 per group. We assume a 20% loss to follow-up at 12 weeks, so the total sample size needed will be 270 × 2/0.8, which is a total of 676 patients recruited from 113 practices across the three recruitment centres (around Southampton, UCL and Liverpool).

### Recruitment {15}

#### Method 1

Where possible, patients who are seen with a new episode of depressive symptoms or disorder will be recruited opportunistically during consultations by participating GPs and NPs in both arms of the study. Patients identified through this method will be given the information sheet by hand, along with a reply slip and a Freepost envelope, and will be asked to contact the study team if they wish to take part.

#### Method 2

Method 1 may be subject to selection bias by the GP/NP; therefore, to guard against this, we will identify all patients presenting with a new episode of depressive symptoms or disorder through weekly searches of practice medical records databases to find patients who were not selected for approach by the GP/NP. In the feasibility trial, both methods were used and 79% of patients were recruited in consultations opportunistically, and 21% through the weekly database searches, but the method used varied by practice, and some practices recruited the majority of patients through the weekly searches.

Our experience gained while recruiting people with depression for previous studies has shown that approximately 120 Read codes are used by GP/NPs, including diagnostic codes (e.g., major depressive disorder) and symptom codes (e.g., low mood). Practices will use the full list for searching their databases weekly. Patients identified through this method will be mailed an information sheet about the study by the practice and asked to contact the study team if they wish to take part, or to decline, using a reply slip and a Freepost envelope. If they do not respond, the research team will have no knowledge of them, maintaining patient confidentiality.

### Assignment of interventions: allocation

#### Sequence generation {16a}

Randomisation will be by computerised sequence generation, and minimisation with a random element using three factors to avoid imbalance between the two arms: practice size (large vs small), location (urban/suburban vs rural), and centre (Southampton vs Liverpool vs UCL).

#### Concealment mechanism {16b}

Randomisation is being carried out by the NIHR Clinical Trials Unit (CTU), Southampton, remote from the research teams recruiting the practices. Notification of allocation is by researcher telephone call to the Unit.

#### Implementation {16c}

The allocation sequence is generated by the CTU, the research teams enrol the participant practices, and the CTU assigns participant practices to the intervention or control arms.

### Assignment of interventions: blinding

#### Who will be blinded {17a}

Blinding of both patients and practitioners in the intervention arm is impossible given the nature of the intervention. Self-report outcome measures are therefore being used to prevent observer rating bias by research team members aware of the patient’s assigned trial arm. The statisticians and health economists analysing the data are being kept blind to the allocation.

#### Procedure for unblinding if needed {17b}

The statisticians and health economists analysing the data will be unblinded to allocation only after all the data have been collected, entered into the database and cleaned.

### Data collection and management

#### Plans for assessment and collection of outcomes {18a}

Data collection will be through face-to-face meetings, but on-line, postal or telephone follow-up will be offered if the researcher is unable to arrange to meet patients face-to-face. Baseline and follow-up assessments will take place either at the patient’s general practice or at their home if they prefer.

Research staff will enter participant data onto study laptop computers and upload them to the study database on return to the University. Study administrative staff will check the data for missing or anomalous values. Data queries will be raised with the recruiting site by the research team.

Qualitative interviews also will be carried out with 15–20 practitioners and 15–20 patients in each arm (total 30–40 of each) to explore their reflections on the conduct of the trial and the potential for implementing the use of PROMs in practice, using NPT as a framework for the initial interview schedules and qualitative analyses. Practitioner/patient dyads will be interviewed as soon as possible after patient assessments at follow-up consultations to explore patient and practitioner recall of interactions within the consultation and to identify variations in the use of PROMs and in usual practitioner care.

#### Plans to promote participant retention and complete follow-up {18b}

Participants will receive a £10 high street shopping voucher at both the 12- and 26-week follow-ups to thank them for their participation in the study. Participants will also receive a £10 high street shopping voucher for taking part in a qualitative interview.

Three attempts will be made to arrange to assess patients face-to-face, by post, or on-line within 4 weeks of the assessment becoming due. Following this further attempts to obtain at least the primary outcome (BDI-II score) and quality of life measure (EQ-5D) over the telephone will be made, if the participant is unable to complete assessment instruments face to face, by post, or on-line.

Patient and practitioner participants will be free to withdraw consent at any time without providing a reason. When withdrawn, patient participants will continue to receive standard clinical care from their practitioner. Follow-up data will continue to be collected (unless the participant has specifically stated that they do not want this to happen).

#### Data management {19}

Participant data will be entered on laptop computers on site and then transferred to electronic databases and stored at the University of Southampton. Data stored will be checked for missing or unusual values (range checks) and checked for consistency within participants over time. Any suspect data will be returned to the researcher or practice in the form of data queries. The CI will be responsible for ensuring the accuracy, completeness and timeliness of the data entered.

#### Confidentiality {27}

Participant data will be pseudo-anonymised by assigning each participant a participant identifier code which will be used to identify the participant during the study and for any participant-specific clarification between the University of Southampton as Sponsor, and the participating general practices.

The Informed Consent Form will specify the participant data to be collected, how it will be managed, and how it might be shared, including the handling of all Patient Identifiable Data (PID) and sensitive PID in adherence to relevant data protection law. Only trained personnel with specific roles assigned will be granted access to the electronic patient data.

Data will be retained at the University of Southampton in accordance with the General Data Protection Regulation (2018). The participants’ medical records and other relevant data may also be reviewed by appropriate qualified personnel independent from the trial team, appointed to audit the study, including representatives of the Competent Authority. Details will remain confidential and participants’ names will not be recorded outside the University.

#### Plans for collection, laboratory evaluation and storage of biological specimens for genetic or molecular analysis in this trial/future use {33}

No biological specimens will be collected.

### Statistical methods

#### Statistical methods for primary and secondary outcomes {20a}

A full and detailed statistical analysis plan will be developed prior to the final analysis of the study. The main features of the statistical analysis plan are as follows:

The primary outcome, that is, the differences at 12 weeks between intervention and controls in depression as measured by the BDI-II, will be analysed using a linear mixed model, adjusting for socio-demographics, baseline depression, anxiety, and clustering, including practice as a random effect. The model will use all the observed data and makes the assumption that missing BDI-II scores are missing completely at random.

Analysis of secondary outcomes, BDI-II at 26 weeks, social functioning, patient satisfaction and quality of life score, will also be conducted using linear regression for continuous outcomes and logistic regression for dichotomous outcomes, again adjusting for socio-demographics, baseline depression, anxiety, and clustering, including practice as a random effect.

A health economic evaluation will be undertaken from a National Health Service and Personal Social Service perspective, with a sensitivity analysis from a societal perspective. The outcome will be expressed as incremental cost per point improvement in the BDI-II clinical outcome, and incremental cost per quality adjusted life year (QALY) gained (cost utility analysis). All items will be costed using appropriate data (e.g., PSSRU NHS and social care reference costs [45]), with informal care costed at minimum wage level. The primary analysis will be at 26 weeks. Personal costs will include patient and carer time off work, personal expenses and travel.

A generalised linear mix model will be used to estimate the differences in costs and QALYs (using the EQ-5D to calculate patient utilities), adjusting for baseline characteristics including depression history, quality of life and sociodemographic factors. Where appropriate, we will estimate incremental cost-effectiveness ratios (ICERs). We will estimate mean values and 95% percentiles using non-parametric bootstrapping, and use these to produce cost-effectiveness acceptability curves (CEACs). Major assumptions in the costing and QALYs analysis will be tested through sensitivity analyses. Modelling of the likely benefit, if any, of using PROMs in practice will include making assumptions about the extra time which would have to be taken for GPs/NPs to administer the initial PROM (rather than the researcher) in the non-trial situation, together with any payments that might have to be made to practices, e.g., through the QOF, to incentivise the use of PROMs.

#### Interim analyses {21b}

No interim analyses are planned. Full details of the analyses to be undertaken will be set out in a statistical analysis plan, to be approved by the independent trial steering committee (TSC).

#### Methods for additional analyses (e.g., subgroup analyses) {20b}

No subgroup analyses are planned. Any post-hoc analyses will be exploratory only.

A qualitative process evaluation will also be conducted. Process evaluation is an important tool for understanding both the dynamics and the outcomes of clinical trials, and Normalization Process Theory NPT [26] is a conceptual toolkit developed for this purpose [46]. NPT focuses on understanding the mechanisms that promote and the factors that inhibit, sense-making, participation, action and monitoring by participants in implementation processes.

The objectives of the process evaluation in the trial are to identify, characterise and explain the perspectives of patient and practitioner participants on the conduct of the trial and to construct a taxonomy of factors affecting both the conduct of the trial and the potential for normalisation of the use of PROMs in everyday practice, outside of the trial situation. The analysis will enable the construction of an implementation framework of barriers and facilitators (patient and health system factors) that need to be taken into account in the use of PROMs in primary care practice.

The qualitative interviews will be transcribed and emerging themes identified through inductive analysis using the constant comparative method [47]. We will draw on insights from the wide range of studies that have employed NPT, giving a basic structure to the topic guide to be written in advance of the interviews. However, we will also work prospectively and inductively to ensure that we identify, characterise and understand (i) disconfirming evidence and deviant cases, and (ii) processes that are not accounted for within NPT.

#### Methods in analysis to handle protocol non-adherence and any statistical methods to handle missing data {20c}

We will examine the structure and pattern of missing data and, if appropriate, will present a sensitivity analysis based on data imputed using a multiple imputation model. Data will be analysed on an intention-to-treat basis.

#### Plans to give access to the full protocol, participant level-data and statistical code {31c}

No plans have been made to share data publicly at present. The trial dissemination group, whose purpose is to oversee the planned outputs from the trial and agree on data sharing arrangements, comprises the CI Tony Kendrick (TK) in Southampton and one co-applicant from each of the other two centres—Liverpool (Chris Dowrick (CD)) and London (Glyn Lewis (GLew)).

### Oversight and monitoring

#### Composition of the coordinating centre and trial steering committee {5d}

TK, CD and GLew lead weekly local study team meetings at Southampton, Liverpool and UCL, respectively, and the overall PROMDEP Trial Management Group (TMG) meets every month by teleconferencing to review progress and give advice on the conduct and management of the study. The TMG includes representatives with expertise in general practice, psychiatry, psychology, sociology, statistics and health economics and is supported by two Patient and Public Involvement (PPI) contributors and CTU staff involved in the day-to-day running of the trial. An independent Trial Steering Committee (TSC) has been set up to oversee trial conduct, consisting of an academic psychologist (chair), academic psychiatrist, statistician, health economist and patient representative.

#### Composition of the data monitoring committee, its role and reporting structure {21a}

An Independent Data Monitoring Committee (IDMC) has also been set up, consisting of an academic general practitioner (chair), statistician and academic psychologist. Although no pre-specified stopping rules have been established, the IDMC will review outcome and safety data regularly during the trial to advise the TSC on continuation of the trial.

#### Adverse event reporting and harms {22}

Any adverse events reported by patients or practitioners will be brought to the attention of the Trial Coordinator and Chief Investigator (CI) or in the absence of the CI, one of the Principal Investigators (PIs). The CI or PI will decide whether or not to inform Sponsor or the Research Ethics Committee (REC), TSC or IDMC. The report will include the event, when the information was reported, assessment of seriousness and likely relationship to participation in the trial. All serious adverse events (SAEs) will be reported to the Chief Investigator and the Trial Coordinator within 24 h of the local site becoming aware of the event. We will record the nature of the event, date of onset, severity, corrective therapies given, outcome, causality (i.e., unrelated, unlikely, possible, probably, definitely) and expectedness. The Chief Investigator will assign the causality and expectedness of the event and the term should be in accordance with the latest version of MedDRA and grades given in accordance with the NCI CTCAE v4.03. Additional information will be provided as soon as possible if the event has not resolved at the time of reporting.

The Chief Investigator or Programme Manager will notify the REC of related and unexpected SAEs occurring during the study according to the following timelines: fatal and life-threatening within 7 days of notification and non-life threatening within 15 days. Adverse events will also be reported to the IDMC, who will advise the TSC about continuation and whether interim analyses are needed. The TSC will work with the IDMC and be kept informed by the CI, PI, or Trial Coordinator. If an extension is requested, the TSC will be responsible for looking into the details as to why this is needed and to give an opinion that will inform the funder (NIHR) and the sponsor (University of Southampton).

#### Frequency and plans for auditing trial conduct {23}

No specific audits are planned. However, participant trial records, medical records and other relevant data may be reviewed by appropriate qualified personnel, including representatives of the Health Research Authority, who are independent from the trial team and appointed to audit the study. Details will remain confidential, and participants’ names will not be recorded outside the University.

#### Plans for communicating important protocol amendments to relevant parties (e.g., trial participants, ethical committees) {25}

Proposed important protocol modifications (e.g., changes to eligibility criteria, outcomes and analyses) will be discussed with the co-investigators before seeking approval from the Health Research Authority and REC, and subsequently, these modifications will be communicated to the trial registry and to any journals where publication is underway).

### Dissemination plans {31a}

The results will be disseminated to participating practices in summary form as well as to academic audiences via publication in peer-reviewed journals and general practice trade publications. We also will publicise our findings through existing primary care networks and patient groups. Summary trial results will be available on the websites of the participating Universities.

## Discussion

If PROMs are helpful in improving patient outcomes for depression even to a small extent, then they are likely to be good value for money, given their low cost. The benefits could be considerable, given that depression is common, disabling and costly.

## Trial status

This paper is based on Version 1.4 of the protocol, dated 4 January 2019 and approved by the REC and HRA on 30 January 2019. Recruitment began on 1 December 2018, and the approximate date when recruitment will be completed is 31 January 2021. The end of the study is defined as the date of the last follow-up visit of the last patient (expected to occur 6 months after the last patient is recruited).

## Data Availability

The anonymised quantitative datasets (but not the qualitative interview data) generated during the current study will be available upon request from Prof. Tony Kendrick (ark1@soton.ac.uk) beginning in 31 October 2022, depending on the types of analyses planned and submission of a peer-reviewed, funded, and ethically approved proposal.

## Abbreviations

BDI-II: Beck Depression Inventory, 2nd edition
CEAC: Cost-effectiveness acceptability curve
CMHD: Common mental health disorder
COM-B: Capability, Opportunity and Motivation Model of Behaviour
CSRI: Client Service Receipt Inventory
CTU: Clinical trials unit
EQ-5D-5 L: EuroQol 5-Dimension, 5-Level Quality of Life Measure
GP: General practitioner
HTA: Health Technology Assessment
HRA: health research authority
ICER: Incremental Cost-effectiveness Ratio
IDMC: Independent data monitoring committee
MCID: Minimal clinically important difference
MISS: Medical Informant Satisfaction Scale
NICE: National Institute for Health and Care Excellence
NIHR: National Institute for Health Research
NP: Nurse practitioner
QALY: Quality-adjusted life year
QOF: Quality and Outcomes Framework
PHQ-9: Patient Health Questionnaire, 9-item version
PID: Patient identifiable data
PROM: Patient-reported Outcome Measure
PSYCHLOPS: Psychological Outcomes Profile Scale
REC: Research ethics committee
TSC: Trial steering committee
UCL: University College London
WSAS: Work and Social Adjustment Scale
